# Meta-Analysis of Long-Term Vitamin D Supplementation on Overall Mortality

**DOI:** 10.1371/journal.pone.0082109

**Published:** 2013-12-03

**Authors:** Yayuan Zheng, Jianhong Zhu, Manru Zhou, Liao Cui, Weimin Yao, Yuyu Liu

**Affiliations:** 1 Department of Pharmacology, Guangdong Medical College, Zhanjiang, China; 2 Institute of Respiratory Disease, Guangdong Medical College, Zhanjiang, China; University of North Carolina at Chapel Hill, United States of America

## Abstract

**Introduction:**

It has been suggested that vitamin D is effective to prevent mortality. However, there is no consistent conclusion that the effects of vitamin D supplementation on all-cause mortality are associated with duration of treatment. We conducted a meta-analysis regarding this issue in an effort to provide a more robust answer.

**Methods:**

A comprehensive search in a number of databases, including MEDLINE, Embase and The Cochrane Central Register of Controlled Trials, was conducted for collecting randomized controlled trials (RCTs) on vitamin D supplementation preventing mortality. Two investigators independently screened the literature according to the inclusive and exclusive criteria and the relative data were extracted. Data analysis was performed by using Review Manager 5.0 software.

**Results:**

Data from forty-two RCT s were included. Vitamin D therapy significantly decreased all-cause mortality with a duration of follow-up longer than 3 years with a RR (95% CI) of 0.94 (0.90–0.98). No benefit was seen in a shorter follow-up periods with a RR (95% CI) of 1.04 (0.97–1.12). Results remain robust after sensitivity analysis. The following subgroups of long-term follow-up had significantly fewer deaths: female only, participants with a mean age younger than 80, daily dose of 800 IU or less, participants with vitamin D insufficiency (baseline 25-hydroxyvitamin D level less than 50 nmol/L) and cholecalciferol therapy. In addition, the combination of vitamin D and calcium significantly reduced mortality and vitamin D alone also had a trend to decrease mortality in a longer time follow up.

**Conclusions:**

The data suggest that supplementation of vitamin D is effective in preventing overall mortality in a long-term treatment, whereas it is not significantly effective in a treatment duration shorter than 3 years. Future studies are needed to identify the efficacy of vitamin D on specific mortality, such as cancer and cardiovascular disease mortality in a long-term treatment duration.

## Introduction

 Vitamin D plays a key role in human health, while the prevalence of vitamin D insufficiency is high, especially among the elderly [1]. It has recently been identified to be associated with skeletal diseases such as osteoporosis [2] and non-skeletal diseases including cancer [3-5], cardiovascular disease [6], and kidney disease [7,8]. Meta-analysis has suggested that low vitamin D baseline levels are associated with increased risks of mortality [9]. This issue is becoming to be paramount importance given the high prevalence of vitamin D deficiency worldwide [10].

 It has been documented that vitamin D supplementation prevents fractures and falls [11,12]. In recent years, several studies on meta-analysis of randomized controlled trials with regard to supplementation of vitamin D on total mortality have been published, which found that vitamin D supplementation reduced total mortality when given together with calcium, but not with vitamin D alone [13-16], and. A Cochrane systematic review found that vitamin D significantly decreased mortality in those with vitamin D insufficiency [15]. However, long-term health effects of vitamin D supplementation still remains unclear.

 To investigate whether the effects of vitamin D supplementation on all-cause mortality are associated with duration of treatment, we undertook a comprehensive systematic database search and meta-analysis to access the effects of vitamin D supplementation on all-cause mortality.

## Materials and Methods

### Search strategy

A literature search was conducted on a number of databases, including Medline, Embase and The Cochrane Central Register of Controlled Trials for the period January 1960 to January 2013, to identify RCTs. Our core search terms were “randomized controlled trial”, “vitamin D”, “vitamin D_2_”, “vitamin D_3_”, “ergocalciferol”, “cholecalciferol”, “mortality”, “death”. We also searched for any additional studies in the reference lists of recent meta-analysis of vitamin D treatment for mortality. Our searches were limited to human trials, and no language or time restriction was applied.

### Eligibility criteria

The preliminary search results were then examed on the basis of the following criteria.

#### Types of studies

Randomized controlled trials evaluating an intervention with vitamin D were identified as part of the review, while review articles, commentaries, letters, observational studies were excluded.

#### Interventions

The intervention group was restricted to vitamin D alone or in combination with calcium treatment; the control group was placebo, no treatment or calcium only therapy. Studies of patients receiving active vitamin D and intramuscular injection of vitamin D were excluded from the review.

#### Outcome

The number of deaths was reported separately for the vitamin D treatment group and the control group. For articles with a large sample size, if the number of deaths was not reported by treatment, we tried to contact the authors to obtain the missing data.

### Data Extraction and Quality Assessment

 Two statisticians independently extracted information from included trials using a standardized form., and then another statisticians verified them. The following information was subtracted from the study: first author, publishing year, sample size, duration, dwelling, intervention, serum 25 (OH) D levels at baseline, and main results (the number of participants who died). Quality assessment of included trials was conducted using the Cochrane Collaboration’s tool [17]. Methodological features most relevant to the control of bias were examined, including random sequence generation, allocation concealment, blinding of participants and personnel, blinding of outcome assessment, incomplete outcome data, selective reporting and other bias [17]. Quality assessment was performed by two independent researchers.

### Data Synthesis and Analysis

 Meta-analysis were undertaken using Review Manager (Version 5.0). The primary outcome was the number of participants who died during follow-up. The pre-planned analysis was vitamin D arm (with or without calcium) versus control arm (placebo, calcium, or no treatment) according to duration of treatment. Mantel-Haenszel method was used to calculate risk ratios (RRs) and their 95% confidence intervals (CI). The I^2^ statistic was used to assess the presence of heterogeneity, which ranges from 0% to 100% [18]. In case of lack of heterogeneity (I^2^ < 50%), fixed-effects model was used to assess the overall estimate, or else random-effects model was chosen. The Begg test [19] and Egger test [20] were used to evaluate the presence of publication bias regarding our primary end points (RR of mortality). A 2-tailed *P* value of less than .05 was considered as statistically significant.

### Sensitivity analysis and subgroup analysis

 A sensitivity analysis was conducted by excluding studies with high risk of bias. Subgroup analysis was conducted only on trials with the duration of treatment at least 3 years or longer. The effect of vitamin D was assessed according to gender (male or female), age group (< 80 years or ≥ 80 years), dose of oral vitamin D daily (≤800 IU or >800 IU), baseline level of 25- hydroxyvitamin D (< 50 nmol per liter or ≥ 50 nmol per liter), type of vitamin D (ergocalciferol or cholecalciferol) and calcium co-administration status (Vitamin D + calcium vs. Calcium, Vitamin D + calcium vs. Placebo, or Vitamin D vs. Placebo), and specific mortality (cancer mortality, cardiovascular mortality)

## Results

### Search Results

**Figure 1:**
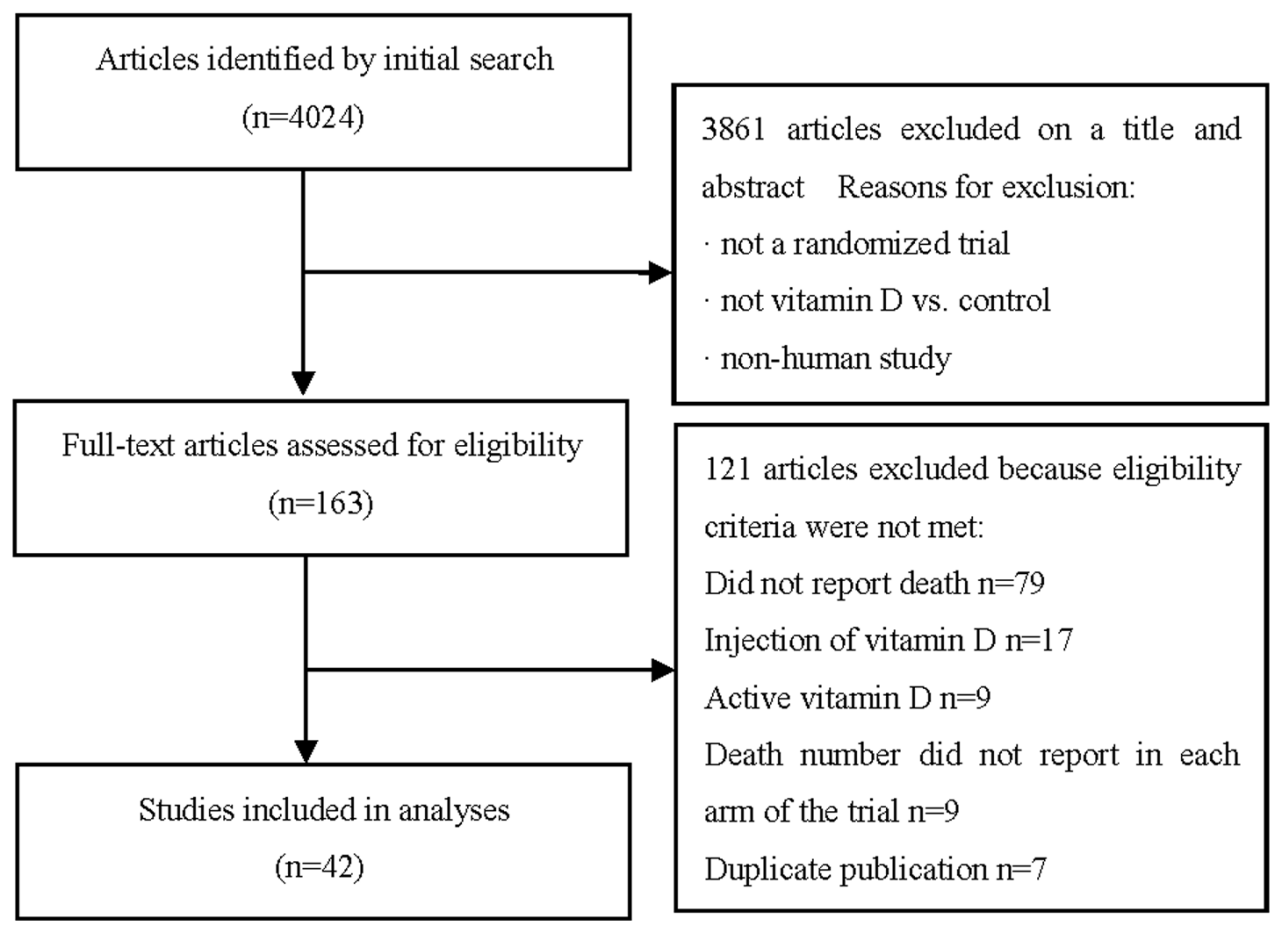
Study flow diagram.

A total of 4,024 unique titles and abstracts were found from initial searches of the electronic database. With the inclusion/exclusion criteria, 3,861 of which were excluded by scrutinizing the titles and abstracts, and 121 articles were further excluded after full text review. A total of 42 RCTs that met inclusion criteria were included in the final analysis [21-62]. The details of study selection flow were explicitly described in Figure 1.

### Study Characteristics

The main characteristics of the included studies are shown in Table 1 (1). Publishing year: The RCTs were published from 1992 to 2012 (2). Treatment duration: 29 RCTs have treatment durations less than 3 years, and the remaining 13 RCTs have treatment durations of 3 years or longer (3). Number of patients: A total of 85,466 patients (42,561 in the vitamin D group and 42,905 in the control group) were included in these 42 RCTs (4). Age of patients: The number of participants in each trial ranged from 46 to 36,282 and mean age of participants ranged from 37 to 89 years, with most participants older than 60 years (5). Vitamin D type and dose: Vitamin D_2_ was used in 10 studies and vitamin D_3_ was used in the remaining 32 studies. Vitamin D_2_ or D_3_ was given as daily doses ranging from 300 to 3,333 IU. Calcium supplementation was used in 26 trials (6). Baseline vitamin D status: 37 trials (80%) reported the baseline vitamin D status of participants based on serum 25-hydroxyvitamin D levels. Participants in 15 trials had baseline 25-hydroxyvitamin D levels at or above cutoff for vitamin D adequacy (50 nmol/l or 20 ng/ml). Participants in the remaining 22 trials had baseline 25-hydroxyvitamin D levels in a range of vitamin D insufficiency (< 50 nmol/l or 20 ng/ml). The other 5 trials did not report the baseline vitamin D status of participants (7). Bias risk: 26 studies had a low risk of bias, and 16 had a high risk of bias (Table 2).

**Table 1:** Characteristics of the included studies.

Study	Population characteristics	Treatment groups	Number of participants	Mean age (years)	Baseline Serum 25 (OH) D levels (mmol/l; mean)	Follow up
Trials have a follow-up of 3 years or longer						
Avenell 2012	Community dwelling, with past low-energy racture	Vit D_3_ 800 IU daily	1343	77	38	5-8 years
		Vit D_3_ 800 IU daily + Ca1000 mg daily	1306	77	38	
		Ca 1000 mg daily	1311	78	38	
		Placebo	1332	77	38	
Bolland 2011	Community-based postmenopausal women	Vit D_3_ 400 IU daily + Ca 1000 mg daily	18176	62	46	7 years
		Placebo	18106	62	48	
Sanders 2010	Ambulatory elderly women at risk for fracture	Vit D_3_ 5000000 IU annually for 3-5 year	1131	77	53	3 years
		Placebo	1125	77	45	
Salovaara 2010	Women aged 65–71 years	Vit D_3_ 800 IU daily + Ca 1000 mg daily	1586	67	50	3 years
		No treatment	1609	67	49	
Zhu 2008	Community-dwelling women aged 70–80 year	Vit D_2_ 1000 IU daily + Ca 1200 mg daily	39	75.4	70.2	5 years
		Ca 1200 mg daily	40	74.1	66.6	
		Placebo	41	74.8	67.3	
Lappe 2007	Healthy postmenopausal white women	Vit D_2_ 1100 IU + Ca 1400-1500 mg daily	446	66.7	71.8	4 years
		Ca 1400-1500 mg daily	445	66.7	71.6	
		Placebo	288	66.7	72.1	
Lyons 2007	Nursing home residents	Initially 100000 IU Vit D_2_ weekly, then 1000 IU Vit D_2_ daily + Ca 600 mg daily	1725	84	80	3 years
		Ca 600 mg daily	1715	84	54	
Aloia 2005	Healthy black postmenopausal women	Vit D_3_ 800 IU daily + Ca 1200 to 1500 mg daily (after 2 years 2000 IU daily)	104	59.9	48.25	3 years
		Ca 1200 to 1500 mg daily	104	61.2	43	
Larsen 2004	Community-dwelling residents	Vit D_3_ 400 IU daily + Ca 1000 mg daily	4957	75	37	42 months
		No treatment	4648	75	33	
Trivedi 2003	Community dwelling individuals	VitD_3_ 100000 IU every 4 months	1345	75	74	5 years
		Placebo	1341	75	53	
Komulainen 1999	Nonosteoporotic, early postmenopausal	Vit D_3_ 300 IU daily (no intake during June-August) for 4 years and 100 IU daily in the5th year + Ca 500 mg daily	112	52.9	NA	5 yeas
		Placebo	115	52.6	NA	
D-Hughes 1997	Healthy, ambulatory eldely older than 65 years	Vit D_3_ 700 IU daily + Ca 500 mg daily	187	71	71.75	3 years
		Placebo	202	72	61.25	
Lips 1996	Elderly living in apartments or homes	VitD_3_ 400 IU daily	1291	80	27	3.5 years
		Placebo	1287	80	26	
Trials have a follow-up of less than 3 years						
Alvarez 2012	Patients with early chronic kidney disease	Vit D 50000 IU weekly for one year	22	62.3	26.7	1 year
		Placebo	24	62.6	32.1	
Lehouck 2012	chronic obstructive pulmonary disease	Vit D_3_ 100000 IU every 4 months for one year	91	68	50	1 year
		Placebo	91	68	50	
TiIDE trial 2012	Patients with type 2 diabetes	Vit D_3_ 1000 IU daily foe one year	607	66.7	NA	6 months
		Placebo	614	66.6	NA	
Wasse 2012	Patients with hemodialysis	Vit D_3_ 200000 IU weekly for 3 weeks	25	49	35.8	53 days
		No treatment	27	52	47.5	
Witham 2010	Older patients with heart failure	Vit D_2_ 100000 IU at 0 and 10 week	53	78.8	20.5	20 weeks
		Placebo	52	80.6	23.7	
Lips 2010	Elderly with vitamin D insufficient	Vit D_3_ 8400 IU weekly for 3 weeks	114	78.5	34.3	16 weeks
		Placebo	112	77.6	35.3	
Wejse 2009	Patients with tuberculosis	Vit D_3_ 100000 IU at 0, 5, 8 month	187	37	77.5	1 year
		Placebo	180	38	79.1	
Chel 2008	Nursing home residents	Vit D_3_ 600 IU daily, or 4200 IU weekly or 180000 IU monthly for 4.5 month	166	84.2	24.9	4.5 months
		Placebo	172	84.2	25.0	
Bjorkman 2008	Aged chronically immobile patients	Vit D_3_ 1200 IU daily	73	83.9	24	6 months
		Vit D_3_ 400 IU daily	77	84.2	21	
		Placebo	68	85.6	23	
Prince 2008	Recruited from emergency department or nursing home	Vit D_2_ 1000 IU daily + Ca 1000 daily	151	77	45	1 year
		Ca 1000 daily	151	77	44	
Burleigh 2007	Rehabilitation wards in an acute geriatric unit	Vit D_3_ 800 IU daily + Ca 1200 daily	101	82	22	1 year
		Ca 1200 daily	104	84	25	
Bolton-Smith 2007	Healthy older women	Vit D_3_ 400 IU daily + Ca1000 daily	62	69.4	62.5	2 years
		Placebo	61	67.8	57	
Broe 2007	Nursing home residents	Vit D_2_ 200-800 IU daily	99	89	48.75	5 months
		Placebo	25	86	50	
Law 2006	Nursing home residents	Vit D_2_ 100000 IU/3 months	1762	85	59	10 months
		Placebo	1955	85	59	
Schleithoff 2006	Patients with congestive heart failure	Vita D_3_ 2000 IU daily + Ca 500 daily for 9 month	61	57	36	15 months
		Ca 500 daily	62	54	38	
Brazier 2005	Ambulatory women aged > 65 years with vitamin D insufficiency	Vit D_3_ 800 IU daily + Ca 1000 daily	95	74.2	18.25	1 year
		Placebo	97	75	17.5	
Flicker 2005	Institutionalized with vitamin D level between 25 and 90 nmol/l	Vit D_3_ initially 100000 IUweekly, then 1000 IU daily + Ca 600 mg daily	313	84	25-90	2 years
		Ca 600 mg daily	312	83	25-90	
Porthouse 2005	Women aged 70 or over with risk factors for hip fracture	Vit D_3_ 800 IU daily + Ca1000 daily	1321	77	NA	25 months
		Leaflet	1993	76.7	NA	
Avenell 2004	Participants had had an osteoporotic fracture within the last 10 years	Vit D_3_ 800 IU daily + Ca1000 daily	99	77	NA	1 year
		No treatment	35	75.6	NA	
Harwood 2004	Elderly women after hip fracture	Vit D_3_ 800 IU+ Ca 1000 mg daily	29	83	29	1 year
		No treatment	35	81	30	
Meier 2004	Healthy adults	VitD_3_ 500 IU+ Ca 500 mg daily	30	55.2	75.25	1.5 years
		No treatment	25	57.9	77	
Cooper 2003	Women who were ≥ 1 y postmenopausal	Vit D_2_ 100000 IU weekly + Ca 1000 daily	93	56.5	81.6	2 years
		Ca 1000 daily	94	56.1	82.6	
Latham 2003	Recruited from geriatric rehabilitation center	Vit D_2_ 300,000 IU/im/once	108	80	38	6 months
		Placebo	114	79	48	
Meyer 2002	Nursing home residents	Vit D_3_ 400 IU daily	569	84	47	2 years
		Placebo	575	85	51	
Chapuy 2002	Elderly ambulatory institutionalized women	Vit D_3_ 800 IU daily + Ca 1200 mg daily	393	85	22.5	2 years
		Placebo	190	85	22.7	
Krieg 1999	Elderly women living in nursing homes	Vit D_3_ 880 IU daily + Ca 1000 mg daily	124	84	29.8	2 years
		No treatment	124	85	29.3	
Bæksgaard 1998	Healthy postmenopausal women	Vit D_3_ 560 IU daily + Ca 1000 mg daily	80	62.9	NA	2 years
		No treatment	80	61.8	NA	
Ooms 1995	Elderly women	VitD_3_ 400 IU daily	177	80.1	27	2 years
		Placebo	171	80.6	25	
Chapuy 1992	Elderly living in apartments or nursing homes	Vit D_3_ 800 IU daily + Ca 1200 mg daily	1634	84	40	2 years
		Placebo	1636	84	32.5	

Vit D, vitamin D; Ca, calcium

**Table 2:** Quality assessment of the included studies.

Study	Random sequence Generation (selection bias)	Allocation Concealment (selection bias)	Blinding of participants and personnel (performance bias)	Blinding of outcome assessment (detection bias)	Incomplete outcome data (attrition bias)	Selective reporting Reporting (reporting bias)	Other bias
Trials have a follow-up of 3 years or longer							
Avenell 2012	L	L	L	L	L	L	L
Bolland 2011	L	L	L	L	L	L	L
Sanders 2010	L	L	L	L	L	L	L
Salovaara 2010	L	L	H	H	L	L	L
Zhu 2008	L	L	L	L	L	L	L
Lappe 2007	L	L	L	L	H	L	L
Lyons 2007	L	L	L	L	L	L	L
Aloia 2005	L	L	L	L	L	L	L
Larsen 2004	U	H	H	H	H	U	H
Trivedi 2003	L	L	L	L	L	L	L
Komulainen 1999	L	L	L	L	L	L	L
D-Hughes 1997	L	L	L	L	L	L	L
Lips 1996	L	L	L	L	L	L	L
Trials have a follow-up of less than 3 years							
Alvarez 2012	L	L	L	L	L	L	L
Lehouck 2012	L	L	L	L	L	L	L
TiIDE trial 2012	L	U	U	U	L	L	H
Wasse 2012	L	L	L	L	L	L	L
Witham 2010	L	L	L	L	L	L	L
Lips 2010	L	L	L	L	L	L	L
Wejse 2009	L	L	L	L	L	L	L
Chel 2008	U	U	U	U	L	L	L
Bjorkman 2008	L	L	L	L	L	L	L
Prince 2008	L	L	L	L	L	L	L
Burleigh 2007	L	L	L	L	L	L	L
Boton-Smith 2007	L	L	L	L	L	L	L
Broe 2007	L	L	L	L	L	L	L
Law 2006	L	H	H	H	L	L	U
Schleithoff 2006	L	L	L	L	L	L	L
Brazier 2005	L	U	U	U	L	L	H
Flicker 2005	L	L	L	L	L	L	L
Porthouse 2005	L	L	H	H	L	U	H
Avenell 2004	L	H	H	H	L	L	H
Harwood 2004	L	L	H	H	L	L	H
Meier 2004	U	U	H	H	L	L	U
Cooper 2003	L	L	L	L	L	L	L
Latham 2003	L	L	L	L	L	L	L
Meyer 2002	H	H	L	L	L	L	H
Chapuy 2002	U	U	U	U	L	L	H
Krieg 1999	U	H	H	H	L	L	L
Bæksgaard 1998	U	U	L	L	L	H	L
Ooms 1995	L	L	L	L	L	L	L
Chapuy 1992	U	U	U	U	L	L	L

### Primary Analysis

**Figure 2:**
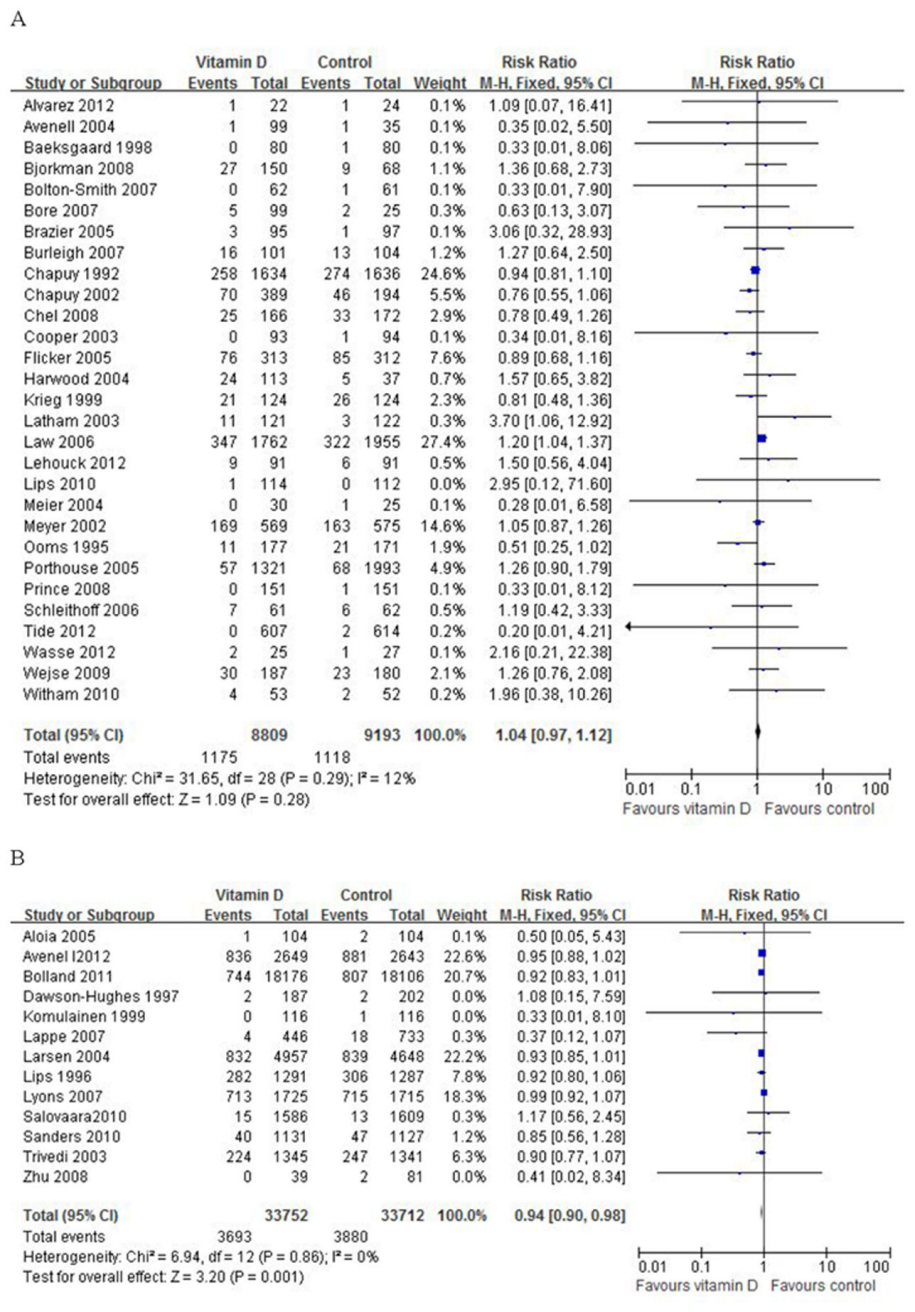
Primary analysis (fixed effect model): A, studies under 3 years; B, studies over 3 years.

 Analysis was performed independently for two categories with follow-up duration either less than 3 years, or 3 years or more. In the category of 29 trials with follow-up less than 3 years, a total of 1,175 (13.3%) participants randomized to the vitamin D group and 1,118 (12.2%) participants randomized to the placebo or no intervention group died. Analysis showed that vitamin D did not significantly decrease all-cause mortality. The risk ratio of mortality for patients treated with vitamin D compared with that of control was 1.04 (95% CI: 0.97–1.12), which was statistically insignificant (P = 0.28), with insignificant heterogeneity (I^2^ = 12%) (Figure 2A). 

 In the second category of 13 trials with follow-up of 3 years or longer, a total of 3,693 (10.9%) participants randomized to the vitamin D group and 3,880 (11.5%) participants randomized to the placebo or no intervention group died. Data analysis showed that vitamin D significantly decreased all-cause mortality with a risk ratio of mortality 0.94 (95% CI: 0.90–0.98), which was statistically significant (P = 0.001), with insignificant heterogeneity (I^2^ = 0%) (Figure 2B).

### Sensitivity analysis

**Figure 3:**
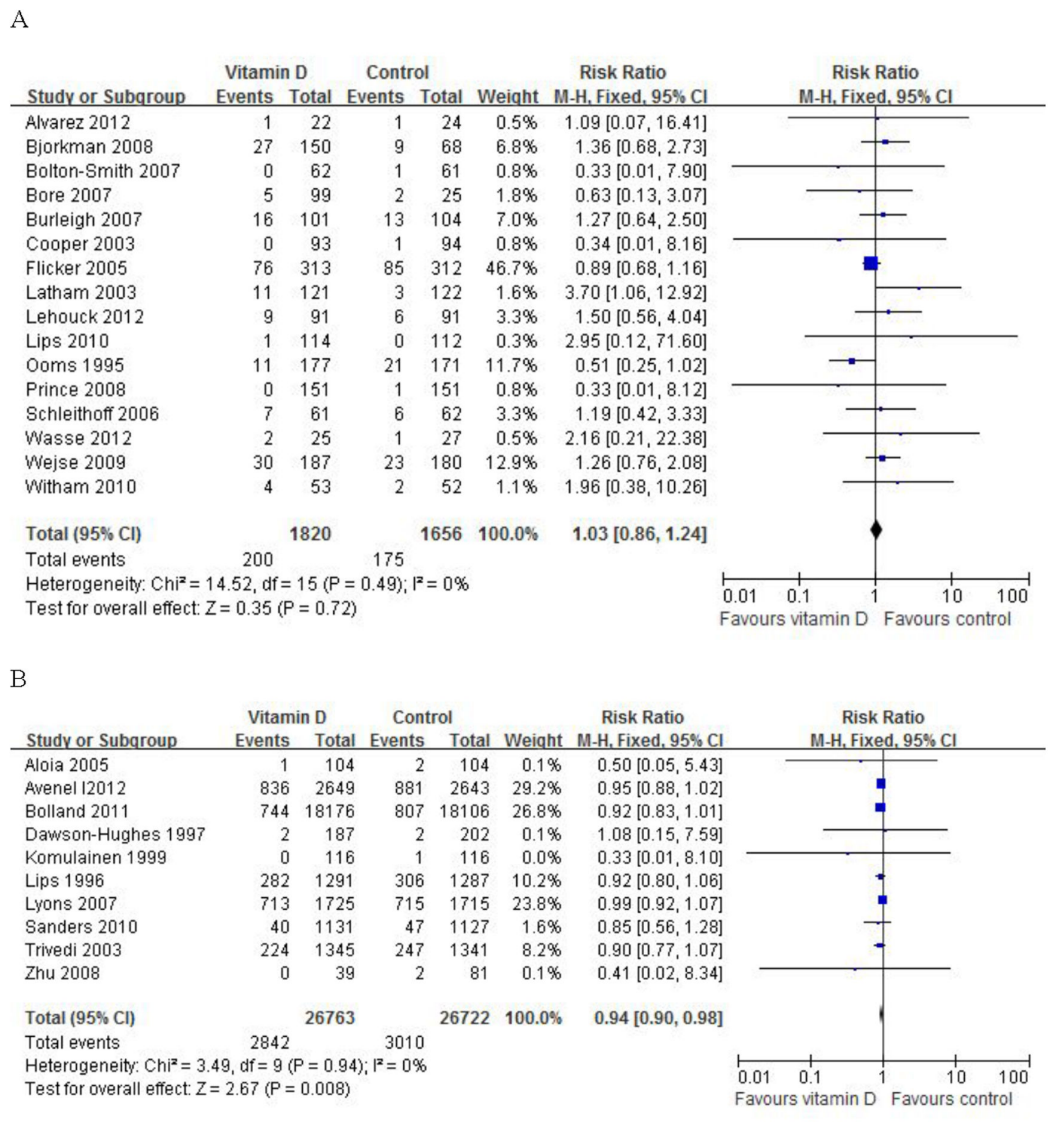
Sensitivity analysis (fixed effect model): A, studies under 3 years; B, studies over 3 years.

 Sensitivity analysis was conducted by excluding the trials that had a high risk of bias, the results remain robust. For studies under 3 years (16 RCTs), the risk ratio of mortality for patients treated with vitamin D compared with control was 1.03 (95% CI: 0.86–1.24), which was not statistically significant (P = 0.72), with insignificant heterogeneity (I^2^ =0%) (Figure 3A). For studies over 3 years (10 RCTs), the risk ratio was 0.94 (95% CI: 0.90–0.98), which was statistically significant (P = 0.008), with insignificant heterogeneity (I^2^ =0%) (Figure 3B). 

### Subgroup analysis of long-term follow-up studies

 In subgroup analysis (Table 3), significantly decreased mortality was seen in women (RR= 0.91; 95% CI: 0.83–1.00). Data on men were limited with only one related trial. Fewer death were found in patients younger than 80 years (RR= 0.93; 95% CI: 0.88–0.97), but not statistically significant in patients aged 80 years or older (RR= 0.97; 95% CI: 0.90–1.04). A dose of 800 IU or less (RR= 0.93; 95% CI: 0.89–0.98) was found to be more favorable than a dose greater than 800 IU (RR= 0.95; 95% CI: 0.89–1.03). Patients with baseline of 25-hydroxyvitamin D level less than 50 nmol/l treated with vitamin D resulted in significant reduction of mortality (RR= 0.93; 95% CI: 0.89–0.98), whereas no effect was seen in patients with baseline of 25-hydroxyvitamin D leve higher than 50 nmol/l (RR= 0.96; 95% CI: 0.89–1.03). Treatment with cholecalciferol (RR= 0.93; 95% CI: 0.89–0.97) was more favorable than with ergocalciferol (RR= 0.98; 95% CI: 0.90–1.06). Vitamin D combined with calcium was effective to reduce mortality when compared to placebo (RR= 0.94; 95% CI: 0.88–0.99), but not significantly effective when compared to calcium (risk ratio 0.97, 95% CI 0.91 to 1.03). The effect of vitamin D alone treatment was statistically insignificant compared to placebo (RR= 0.93; 95% CI: 0.86–1.00). Vitamin D treatment significantly reduced the cancer mortality (RR= 0.88; 95% CI: 0.79–0.98), but did not decrease cardiovascular mortality (RR= 0.91; 95% CI: 0.81–1.02). 

**Table 3:** Subgroup benefits at the longer duration of Vitamin D, as compared with control group (Trial level data).

Subgroup	No. of participants		No. of death		Risk ratio (95% CI)	P Value	I^2^, %
	Vitamin D group	Control group	Vitamin D group	Control group			
Gender							
Male only	1019	1018	199	220	0.88 (0.73–1.07)	0.19	0
Female only	22111	22401	831	919	0.91 (0.83–1.00)	0.04	0
Age							
<80 yr	30736	30710	2698	2859	0.93 (0.88–0.97)	0.002	0
≥80 yr	3016	3002	995	1021	0.97 (0.90–1.04)	0.39	0
Dose of oral vitamin D, IU	P=0.61						
≤800	29066	28715	2712	2851	0.93 (0.89–0.98)	0.003	0
>800	4686	4997	981	1029	0.95 (0.89–1.03)	0.20	19
Baseline 25-hydroxyvitamin D*							
<50	27177	26788	2695	2835	0.93 (0.89–0.98)	0.003	0
≥50	6459	6808	998	1044	0.96 (0.89–1.03)	0.23	0
Type of vitamin D							
Ergocalciferol	2210	2529	717	735	0.98 (0.90–1.06)	0.59	45
Cholecalciferol	31542	31183	2976	3145	0.93 (0.89–0.97)	0.001	0
Calcium coadministration status							
Vitamin D + calcium *vs.* calcium	3174	3170	1129	1164	0.97 (0.91–1.03)	0.32	0
Vitamin D + calcium *vs.* placebo	26367	26054	2008	2098	0.94 (0.88–0.99)	0.02	0
Vitamin D *vs.* placebo	5110	5087	967	1034	0.93 (0.86–1.00)	0.06	0
Specific mortality							
Cancer mortality	22170	22090	558	632	0.88 (0.79–0.98)	0.03	0
Cardiovascular mortality	22170	22090	489	537	0.91 (0.81–1.02)	0.11	0

* Based on the reported mean baseline level, irrespective of type of vitamin D assay, in a sample of study participants

### Publication bias

No evidence of publication bias was detected for the risk ratio of mortality in this study by either Begg or Egger’s test. For studies under 3 years, Begg’s test P= 0.837, Egger’s test P= 0.623; For studies over 3 years, Begg’s test P= 0.059, Egger’s test P= 0.055.

## Discussion

 We conducted a systematic review and meta-analysis to evaluate the best available research evidence regarding vitamin D supplementation on overall mortality. A total of 42 RCTs were included in the present study, quality assessment suggested that the overall study quality was fair and no significant publication bias was detected. Our results demonstrates that vitamin D supplementation longer than 3 years leads to a significant reduction on overall mortality. When trials with a high risk of bias excluded in the sensitive analysis, the results remain robust. The effect of vitamin D on mortality reduction was significant in several subgroups of individuals: female patients, participants with a mean age younger than 80, dose of 800 IU or less, participants with vitamin D insufficiency (baseline 25-hydroxyvitamin D level less than 50 nmol/L) and cholecalciferol therapy. In addition, compared with placebo, vitamin D in combination with calcium significantly reduced mortality.

 Our findings confirmed those in an earlier Cochrane systematic review [15] on the effect of vitamin D treatment on overall mortality, which showed that participants with vitamin D insufficiency (25-hydroxyvitamin D level less than 20 ng/ml) decreased the overall mortality significantly, and indicated that cholecalciferol therapy was more favorable than ergocalciferol and that vitamin D as daily doses of 800 IU or less was more favorable than daily doses more than 800 IU. In contrast with two meta-analysis [13,16], which compared daily dose of 800 IU or greater with that less than 800 IU and suggested that daily dose of vitamin D did not differ in the effect on the outcome, our analysis indicated that the beneficial effect of vitamin D is clearly observed in the low daily dose. One explanation may be that several included trials [23,27,30] used intermittent and high dose of vitamin D, which has been suggested less likely to have a benefit, or to even have a negative effect among the elderly [23]. Consumption of intermittent and high dose of vitamin D leads to high concentrations of plasma 25-hydroxyvitamin D. Michaëlsson et al [63] concluded that both high and low concentrations of plasma 25-hydroxyvitamin D were associated with elevated risks of overall and cancer mortality.

 A previous meta-analysis conducted by Autier et al [13] suggested that no relationship was found with duration of vitamin D supplement. In contrast, our results indicated that vitamin D supplementation significantly reduced the overall mortality when duration was longer than 3 years compared with that of control. However, no benefit was seen in those with durations less than 3 years. Additionally, two meta-analyses of RCTs with vitamin D treatment on falls also reported that patients benefit from vitamin D supplementation in a longer time duration [64,65]. 

 Our results indicated that vitamin D was effective in reducing mortality among female patients. There was a lack of evidence to draw a conclusion of vitamin D’s influence on male patients with only one identified trial collected death data by subgroup of gender. We concluded that vitamin D may decrease mortality in patients younger than 80 years old, but not in patients aged 80 years or older. However, no statistically significant difference was found for risk ratio of overall mortality between the two age groups (*P*= 0.59). This results support an early meta-analysis of vitamin D treatment on falls, which indicated that participants with a mean age younger than 80 benefited from vitamin D supplementation [64].

 Several previous meta-analyses suggested that vitamin D supplementation reduced all-cause mortality when given together with calcium, but did not support an effect of vitamin D alone treatment [14-16]. In contrast, our results suggested that vitamin D combined with calcium reduced all-cause mortality significantly when compared with placebo (RR= 0.94; 95% CI: 0.88–0.99), but the effect was insignificantly when compared with calcium therapy (RR= 0.97; 95% CI: 0.91–1.03). Vitamin D alone had a trend to decrease mortality (RR= 0.93; 95% CI: 0.86–1.00) when administrated in a long time. It may indicate that calcium therapy does not increase risk of death [66]. Whether vitamin D given together with calcium is more beneficial than calcium alone treatment needs more RCTs to be clarified.

 There is not sufficient evidence to draw conclusions of the effect of vitamin D on specific mortality with only 3 trials collected mortality data in a rigorous fashion. Vitamin D may have a beneficial effect on cancer related mortality. But it needs more RCTs to better understand the effect of vitamin D on cancer. Meta-analyses of cohort studies have suggested that vitamin D intake was associated with reduced risk of colorectal cancer [67], breast cancer [68] but not prostate cancer [69]. Vitamin D had no significant effect on cardiovascular disease mortality. A growing number of literature suggest that low levels of vitamin D are associated with cardiovascular disease risk [70-74]. A limited number of interventional studies that investigated the effects of vitamin D supplementation on cardiovascular disease risk showed mixed results [75-78]. The effect of vitamin D on cardiovascular diseases remains to be identified. 

 The mechanism of vitamin D benefit on overall mortality is not clear. Both forms of vitamin D (D_2_ and D_3_) are converted to 25-hydroxyvitamin [25(OH)D] in the liver, and then hydroxylated to 1,25-dihydroxyvitamin D in the kidney [79]. 1,25(OH)_2_D is the only biologically active form of vitamin D, which increases calcium absorption and bone formation to maintain bone health, regulate blood pressure and insulin production, prevent heart disease, regulate immune function to prevent diabetes and autoimmune disease, regulate cell growth to prevent cancer [80,81]. 

 Vitamin D insufficiency (< 50 nmol/L) has now been linked to a broad spectrum of human diseases from cancer to cardiovascular to autoimmune conditions [82]. Though vitamin D can acquire through cutaneous synthesis after sunlight exposure and nutrition [83], it is often not sufficient to reach the required levels of vitamin D, especially in patients with osteoporosis and fracture risk [84]. In that case, supplementation of vitamin D is required, in order to prevent vitamin D insufficiency and associated adverse outcomes.

 Similar to other meta-analyses, our review has several limitations. First, though extensive searches were made, there were no data of Hispanic or Orientals. Second, most of the participants in the present study were older women, the effects of vitamin D on mortality in younger, healthy persons and in males are still inconclusive. Third, the overall RR (95% CI) effect was modest and could be the result of chance alone. Fourth, there were only 13 trials that have durations of follow up longer than 3 years.

 In conclusion, our results implicated that long-term supplementation of vitamin D may have a beneficial effect on overall mortality, especially in patients with vitamin D insufficiency and younger than 80 years. Vitamin D in a dose of 800 IU daily or less was found to be more favorable than a dose greater than 800 IU and treatment with cholecalciferol was more favorable than ergocalciferol. Future studies are needed to test the efficacy of vitamin D on specific mortality, such as cancer and cardiovascular disease mortality in a long-term treatment duration.

## Supporting Information

Checklist S1 **PRISMA checklist.**
(DOC)

## References

[1] Wahl DA, Cooper C, Ebeling PR, Eggersdorfer M, Hilger J et al. (2012) A global representation of vitamin D status in healthy populations. Arch Osteoporos 7: 155–172. doi:10.1007/s11657-012-0093-0. PubMed: 23225293. 23225293

[2] Rovner AJ, Miller RS (2008) Vitamin D deficiency and insufficiency in children with osteopenia or osteoporosis. Pediatrics 122: 907-908. doi:10.1542/peds.2008-1743. PubMed: 18829822. 18829822

[3] Ma Y, Zhang P, Wang F, Yang J, Liu Z, et aal (2011) Association between vitamin D and risk of colorectal cancer: a systematic review of prospective studies. J Clin Oncol 29: 3775–3782. doi:10.1200/JCO.2011.35.7566. PubMed: 21876081. 21876081

[4] Abbas S, Chang-Claude J, Linseisen J (2009) Plasma 25-hydroxyvitamin D and premenopausal breast cancer risk in a German case-control study. Int J Cancer 124: 250–255. doi:10.1002/ijc.23904. PubMed: 18839430. 18839430

[5] Kilkkinen A, Knekt P, Heliövaara M, Rissanen H, Marniemi J et al. (2008) Vitamin D status and the risk of lung cancer: a cohort study in Finland. Cancer Epidemiol Biomarkers Prev 17: 3274–3278. doi:10.1158/1055-9965.EPI-08-0199. PubMed: 18990771. 18990771

[6] Artaza JN, Mehrotra R, Norris KC (2009) Vitamin D and the cardiovascular system. Clin J Am Soc Nephrol 4: 1515-1522. doi:10.2215/CJN.02260409. PubMed: 19696220. 19696220

[7] Ravani P, Malberti F, Tripepi G, Pecchini P, Cutrupi S et al. (2009) Vitamin D levels and patient outcome in chronic kidney disease. Kidney Int 75: 88–95. doi:10.1038/ki.2008.501. PubMed: 18843258. 18843258

[8] Mathieu C, Gysemans C, Giulietti A, Bouillon R (2005) Vitamin D and diabetes. Diabetologia 48: 1247-1257. doi:10.1007/s00125-005-1802-7. PubMed: 15971062. 15971062

[9] Melamed ML, Michos ED, Post W, Astor B (2008) 25-hydroxyvitamin D levels and the risk of mortality in the general population. Arch Intern Med 168: 1629–1630. doi:10.1001/archinte.168.15.1629. PubMed: 18695076. 18695076 PMC2677029

[10] Mithal A, Wahl DA, Bonjour JP, Burckhardt P, Dawson-Hughes B et al. (2009) Global vitamin D status and determinants of hypovitaminosis D. Osteoporos Int 20: 1807–1820. doi:10.1007/s00198-009-0954-6. PubMed: 19543765. 19543765

[11] Bischoff-Ferrari HA, Dawson-Hughes B, Staehelin HB, Orav JE, Stuck AE et al. (2009) Fall prevention with supplemental and active forms of vitamin D: a meta-analysis of randomised controlled trials. BMJ 339: b3692. doi:10.1136/bmj.b3692. PubMed: 19797342. 19797342 PMC2755728

[12] Bischoff-Ferrari HA, Willett WC, Orav EJ, Lips P, Meunier PJ et al. (2012) A pooled analysis of vitamin D dose requirements for fracture prevention. N Engl J Med 367: 40-49. doi:10.1056/NEJMoa1109617. PubMed: 22762317. 22762317

[13] Autier P, Gandini S (2007) Vitamin D supplementation and total mortality: a meta-analysis of randomized controlled trials. Arch Intern Med 167: 1730–1737. doi:10.1001/archinte.167.16.1730. PubMed: 17846391. 17846391

[14] Chung M, Balk EM, Brendel M, Ip S, Lau J et al. (2009) Vitamin D and calcium: a systematic review of health outcomes. Evid Rep Technol Assess (Full Rep) 183: 1–420. PubMed: 20629479. PMC4781105 20629479

[15] Bjelakovic G, Gluud LL, Nikolova D, Whitfield K, Wetterslev J et al. (2011) Vitamin D supplementation for prevention of mortality in adults. Cochrane Database Syst Rev 7: CD007470:CD007470 PubMed: 21735411. 10.1002/14651858.CD007470.pub2 21735411

[16] Rejnmark L, Avenell A, Masud T, Anderson F, Meyer HE et al. (2012) Vitamin D with calcium reduces mortality: patient level pooled analysis of 70,528 patients from eight major vitamin D trials. J Clin Endocrinol Metab 97: 2670-2681. doi:10.1210/jc.2011-3328. PubMed: 22605432. 22605432 PMC3410276

[17] Higgins JP, Altman DG, Gøtzsche PC, Jüni P, Moher D et al. (2011) The Cochrane Collaboration's tool for assessing risk of bias in randomised trials. BMJ 343: d5928. doi:10.1136/bmj.d5928. PubMed: 22008217. 22008217 PMC3196245

[18] Higgins JPT, Green S, Cochrane Collaboration (2008) Cochrane Handbook for Systematic Reviews of Interventions. Chichester, England; Hoboken, NJ:Wiley-Blackwell.

[19] Begg CB, Mazumdar M (1994) Operating characteristics of a rank correlation test for publication bias. Biometrics 50(4): 1088–1101. doi:10.2307/2533446. PubMed: 7786990. 7786990

[20] Egger M, Davey SG, Schneider M, Minder C (1997) Bias in meta-analysis detected by a simple, graphical test. BMJ 315: 629–634. doi:10.1136/bmj.315.7109.629. PubMed: 9310563. 9310563 PMC2127453

[21] Avenell A, MacLennan GS, Jenkinson DJ, McPherson GC, McDonald AM et al. (2012) Long-term follow-up for mortality and cancer in a randomized placebo-controlled trial of vitamin D(3) and/or calcium (RECORD trial). J Clin Endocrinol Metab 97: 614-622. doi:10.1210/jc.2011-1309. PubMed: 22112804. 22112804

[22] Bolland MJ, Grey A, Gamble GD, Reid IR (2011) Calcium and vitamin D supplements and health outcomes: a reanalysis of the Women’s Health Initiative (WHI) limited-access data set. Am J Clin Nutr 94: 1144-1149. doi:10.3945/ajcn.111.015032. PubMed: 21880848. 21880848 PMC3173029

[23] Sanders KM, Stuart AL, Williamson EJ, Simpson JA, Kotowicz MA et al. (2010) Annual high-dose oral vitamin D and falls and fractures in older women: a randomized controlled trial. JAMA 303: 1815-1822. doi:10.1001/jama.2010.594. PubMed: 20460620. 20460620

[24] Salovaara K, Tuppurainen M, Kärkkäinen M, Rikkonen T, Sandini L et al. (2010) Effect of vitamin D(3) and calcium on fracture risk in 65- to 71-year-old women: a population-based 3-year randomized, controlled trial--the OSTPRE-FPS. J Bone Miner Res 25: 1487-1495. doi:10.1002/jbmr.48. PubMed: 20200964. 20200964

[25] Zhu K, Devine A, Dick IM, Wilson SG, Prince RL (2008) Effects of calcium and vitamin D supplementation on hip bone mineral density and calcium-related analytes in elderly ambulatory Australian women: a five-year randomized controlled trial. J Clin Endocrinol Metab 93: 743–749. PubMed: 18089701. 18089701 10.1210/jc.2007-1466

[26] Lappe JM, Travers-Gustafson D, Davies KM, Recker RR, Heaney RP (2007) Vitamin D and calcium supplementation reduces cancer risk: results of a randomized trial. Am J Clin Nutr 85: 1586–1591. PubMed: 17556697. 17556697 10.1093/ajcn/85.6.1586

[27] Lyons RA, Johansen A, Brophy S, Newcombe RG, Phillips CJ et al. (2007) Preventing fractures among older people living in institutional care: a pragmatic randomised double blind placebo controlled trial of vitamin D supplementation. Osteoporos Int 18: 811–818. doi:10.1007/s00198-006-0309-5. PubMed: 17473911. 17473911

[28] Aloia JF, Talwar SA, Pollack S, Yeh J (2005) A randomized controlled trial of vitamin D3 supplementation in African American women. Arch Intern Med 165: 1618–1623. doi:10.1001/archinte.165.14.1618. PubMed: 16043680. 16043680 PMC1464166

[29] Larsen ER, Mosekilde L, Foldspang A (2004) Vitamin D and calcium supplementation prevents osteoporotic fractures in elderly community dwelling residents: a pragmatic population-based 3-year intervention study. J Bone Miner Res 19: 370–378. PubMed: 15040824. 15040824 10.1359/JBMR.0301240

[30] Trivedi DP, Doll R, Khaw KT (2003) Effect of four monthly oral vitamin D3 (cholecalciferol) supplementation on fractures and mortality in men and women living in the community: randomised double blind controlled trial. BMJ 326: 469. doi:10.1136/bmj.326.7387.469. PubMed: 12609940. 12609940 PMC150177

[31] Komulainen M, Kröger H, Tuppurainen MT, Heikkinen AM, Alhava E et al. (1999) Prevention of femoral and lumbar bone loss with hormone replacement therapy and vitamin D3 in early postmenopausal women: a population-based 5-year randomized trial. J Clin Endocrinol Metab 84: 546–552. doi:10.1210/jc.84.2.546. PubMed: 10022414. 10022414

[32] Dawson-Hughes B, Harris SS, Krall EA, Dallal GE (1997) Effect of calcium and vitamin D supplementation on bone density in men and women 65 years of age or older. N Engl J Med 337: 670–676. doi:10.1056/NEJM199709043371003. PubMed: 9278463. 9278463

[33] Lips P, Graafmans WC, Ooms ME, Bezemer PD, Bouter LM (1996) Vitamin D supplementation and fracture incidence in elderly persons. A randomized, placebo-controlled clinical trial. Ann Intern Med 124: 400–406. doi:10.7326/0003-4819-124-4-199602150-00003. PubMed: 8554248. 8554248

[34] Alvarez JA, Law J, Coakley KE, Zughaier SM, Hao L et al. (2012) High-dose cholecalciferol reduces parathyroid hormone in patients with early chronic kidney disease: a pilot, randomized, double-blind, placebo-controlled trial. Am J Clin Nutr 96: 672-679. doi:10.3945/ajcn.112.040642. PubMed: 22854402. 22854402 PMC3417221

[35] Lehouck A, Mathieu C, Carremans C, Baeke F, Verhaegen J et al. (2012) High doses of vitamin D to reduce exacerbations in chronic obstructive pulmonary disease. Ann Intern Med 156: 105-114. doi:10.7326/0003-4819-156-2-201201170-00004. PubMed: 22250141. 22250141

[36] Punthakee Z, Bosch J, Dagenais G, Diaz R, Holman R et al. (2012) Design, history and results of the Thiazolidinedione Intervention with vitamin D Evaluation (TIDE) randomised controlled trial. Diabetologia 55: 36-45. doi:10.1007/s00125-011-2357-4. PubMed: 22038523. 22038523

[37] Wasse H, Huang R, Long Q, Singapuri S, Raggi P et al. (2012) Efficacy and safety of a short course of very-high-dose cholecalciferol in hemodialysis. Am J Clin Nutr 95: 522-528. doi:10.3945/ajcn.111.025502. PubMed: 22237061. 22237061 PMC3260077

[38] Witham MD, Crighton LJ, Gillespie ND, Struthers AD, McMurdo ME (2010) The effects of vitamin D supplementation on physical function and quality of life in older patients with heart failure a randomized controlled trial. Circ Heart Fail 3: 195-201. doi:10.1161/CIRCHEARTFAILURE.109.907899. PubMed: 20103775. 20103775

[39] Lips P, Binkley N, Pfeifer M, Recker R, Samanta S et al. (2010) Once-weekly dose of 8400 IU vitamin D(3) compared with placebo: effects on neuromuscular function and tolerability in older adults with vitamin D insufficiency. Am J Clin Nutr 91: 985–991. doi:10.3945/ajcn.2009.28113. PubMed: 20130093. 20130093

[40] Wejse C, Gomes VF, Rabna P, Gustafson P, Aaby P et al. (2009) Vitamin D as supplementary treatment for tuberculosis: a double-blind, randomized, placebocontrolled trial. Am J Respir Crit Care Med 179: 843–850. doi:10.1164/rccm.200804-567OC. PubMed: 19179490. 19179490

[41] Chel V, Wijnhoven HA, Smit JH, Ooms M, Lips P (2008) Efficacy of different doses and time intervals of oral vitamin D supplementation with or without calcium in elderly nursing home residents. Osteoporos Int 19: 663–671. doi:10.1007/s00198-007-0465-2. PubMed: 17874029. 17874029 PMC2277446

[42] Björkman M, Sorva A, Risteli J, Tilvis R (2008) Vitamin D supplementation has minor effects on parathyroid hormone and bone turnover markers in vitamin D-deficient bedridden older patients. Age Ageing 37: 25–31. PubMed: 17965037. 17965037 10.1093/ageing/afm141

[43] Prince RL, Austin N, Devine A, Dick IM, Bruce D et al. (2008) Effects of ergocalciferol added to calcium on the risk of falls in elderly high-risk women. Arch Intern Med 168: 103–108. doi:10.1001/archinternmed.2007.31. PubMed: 18195202. 18195202

[44] Burleigh E, McColl J, Potter J (2007) Does vitamin D stop inpatients falling? A randomised controlled trial. Age Ageing 36: 507–513. doi:10.1093/ageing/afm087. PubMed: 17656420. 17656420

[45] Bolton-Smith C, McMurdo ME, Paterson CR, Mole PA, Harvey JM et al. (2007) Two-year randomized controlled trial of vitamin K1 (phylloquinone) and vitamin D3 plus calcium on the bone health of older women. J Bone Miner Res 22: 509–519. doi:10.1359/jbmr.070116. PubMed: 17243866. 17243866

[46] Broe KE, Chen TC, Weinberg J, Bischoff-Ferrari HA, Holick MF et al. (2007) A higher dose of vitamin d reduces the risk of falls in nursing home residents: a randomized, multiple-dose study. J Am Geriatr Soc 55: 234–239. doi:10.1111/j.1532-5415.2007.01048.x. PubMed: 17302660. 17302660

[47] Law M, Withers H, Morris J, Anderson F (2006) Vitamin D supplementation and the prevention of fractures and falls: results of a randomised trial in elderly people in residential accommodation. Age Ageing 35: 482–486. doi:10.1093/ageing/afj080. PubMed: 16641143. 16641143

[48] Schleithoff SS, Zittermann A, Tenderich G, Berthold HK, Stehle P et al. (2006) Vitamin D supplementation improves cytokine profiles in patients with congestive heart failure: a double-blind, randomized, placebo-controlled trial. Am J Clin Nutr 83: 754–759. PubMed: 16600924. 16600924 10.1093/ajcn/83.4.754

[49] Brazier M, Grados F, Kamel S, Mathieu M, Morel A et al. (2005) Clinical and laboratory safety of one year’s use of a combination calcium + vitamin D tablet in ambulatory elderly women with vitamin D insufficiency: results of a multicenter, randomized, double-blind, placebo controlled study. Clin Ther 27: 1885–1893. doi:10.1016/j.clinthera.2005.12.010. PubMed: 16507374. 16507374

[50] Flicker L, MacInnis RJ, Stein MS, Scherer SC, Mead KE et al. (2005) Should older people in residential care receive vitamin D to prevent falls? Results of a randomized trial. J Am Geriatr Soc 53: 1881–1888. doi:10.1111/j.1532-5415.2005.00468.x. PubMed: 16274368. 16274368

[51] Porthouse J, Cockayne S, King C, Saxon L, Steele E et al. (2005) Randomised controlled trial of calcium and supplementation with cholecalciferol (vitamin D3) for prevention of fractures in primary care. BMJ 330: 1003. doi:10.1136/bmj.330.7498.1003. PubMed: 15860827. 15860827 PMC557150

[52] Avenell A, Grant AM, McGee M, McPherson G, Campbell MK et al. (2004) The effects of an open design on trial participant recruitment, compliance and retention--a randomized controlled trial comparison with a blinded, placebo-controlled design. Clin Trials 1: 490–498. doi:10.1191/1740774504cn053oa. PubMed: 16279289. 16279289

[53] Harwood RH, Sahota O, Gaynor K, Masud T, Hosking DJ et al. (2004) A randomised, controlled comparison of different calcium and vitamin D supplementation regimens in elderly women after hip fracture: The Nottingham Neck of Femur (NONOF) Study. Age Ageing 33: 45–51. doi:10.1093/ageing/afh002. PubMed: 14695863. 14695863

[54] Meier C, Woitge HW, Witte K, Lemmer B, Seibel MJ (2004) Supplementation with oral vitamin D3 and calcium during winter prevents seasonal bone loss: a randomized controlled open-label prospective trial. J Bone Miner Res 19: 1221–1230. doi:10.1359/JBMR.040511. PubMed: 15231008. 15231008

[55] Cooper L, Clifton-Bligh PB, Nery ML, Figtree G, Twigg S et al. (2003) Vitamin D supplementation and bone mineral density in early postmenopausal women. Am J Clin Nutr 77: 1324–1329. PubMed: 12716689. 12716689 10.1093/ajcn/77.5.1324

[56] Latham NK, Anderson CS, Lee A, Bennett DA, Moseley A et al. (2003) A randomized, controlled trial of quadriceps resistance exercise and vitamin D in frail older people: the Frailty Interventions Trial in Elderly Subjects (FITNESS). J Am Geriatr Soc 51: 291–299. doi:10.1046/j.1532-5415.2003.51101.x. PubMed: 12588571. 12588571

[57] Meyer HE, Smedshaug GB, Kvaavik E, Falch JA, Tverdal A et al. (2002) Can vitamin D supplementation reduce the risk of fracture in the elderly? A randomized controlled trial. J Bone Miner Res 17: 709–715. doi:10.1359/jbmr.2002.17.4.709. PubMed: 11918228. 11918228

[58] Chapuy MC, Pamphile R, Paris E, Kempf C, Schlichting M et al. (2002) Combined calcium and vitamin D3 supplementation in elderly women: confirmation of reversal of secondary hyperparathyroidism and hip fracture risk: the Decalyos II study. Osteoporos Int 13: 257–264. doi:10.1007/s001980200023. PubMed: 11991447. 11991447

[59] Krieg MA, Jacquet AF, Bremgartner M, Cuttelod S, Thiébaud D et al. (1999) Effect of supplementation with vitamin D3 and calcium on quantitative ultrasound of bone in elderly institutionalized women: a longitudinal study. Osteoporos Int 9: 483–488. doi:10.1007/s001980050174. PubMed: 10624454. 10624454

[60] Baeksgaard L, Andersen KP, Hyldstrup L (1998) Calcium and vitamin D supplementation increases spinal BMD in healthy, postmenopausal women. Osteoporos Int 8: 255–260. doi:10.1007/s001980050062. PubMed: 9797910. 9797910

[61] Ooms ME, Roos JC, Bezemer PD, van der Vijgh WJ, Bouter LM et al. (1995) Prevention of bone loss by vitamin D supplementation in elderly women: a randomized doubleblind trial. J Clin Endocrinol Metab 80: 1052–1058. doi:10.1210/jc.80.4.1052. PubMed: 7714065. 7714065

[62] Chapuy MC, Arlot ME, Duboeuf F, Brun J, Crouzet B et al. (1992) Vitamin D3 and calcium to prevent hip fractures in the elderly women. N Engl J Med 327: 1637–1642. doi:10.1056/NEJM199212033272305. PubMed: 1331788. 1331788

[63] Michaëlsson K, Baron JA, Snellman G, Gedeborg R, Byberg L et al. (2010) Plasma vitamin D and mortality in older men: a community-based prospective cohort study. Am J Clin Nutr 92: 841-848. doi:10.3945/ajcn.2010.29749. PubMed: 20720256. 20720256

[64] Kalyani RR, Stein B, Valiyil R, Manno R, Maynard JW et al. (2010) Vitamin D treatment for the prevention of falls in older adults: systematic review and meta-analysis. J Am Geriatr Soc 58: 1299-1310. doi:10.1111/j.1532-5415.2010.02949.x. PubMed: 20579169. 20579169 PMC3125705

[65] Bischoff-Ferrari HA, Dawson-Hughes B, Staehelin HB, Orav JE, Stuck AE et al. (2009) Fall prevention with supplemental and active forms of vitamin D: a meta-analysis of randomised controlled trials. BMJ 339: b3692. doi:10.1136/bmj.b3692. PubMed: 19797342. 19797342 PMC2755728

[66] Uusi-Rasi K, Kärkkäinen MU, Lamberg-Allardt CJ (2013) Calcium intake in health maintenance - a systematic review. Food. Nutr Res 57. doi:10.3402/fnr.v57i0.21082. PMC3657072 23687486

[67] Touvier M, Chan DS, Lau R, Aune D, Vieira R et al. (2011) Meta-analyses of vitamin D intake, 25-hydroxyvitamin D status, vitamin D receptor polymorphisms, and colorectal cancer risk. Cancer Epidemiol Biomarkers Prev 20: 1003-1016. doi:10.1158/1055-9965.EPI-10-1141. PubMed: 21378269. 21378269

[68] Chen P, Hu P, Xie D, Qin Y, Wang F et al. (2010) Meta-analysis of vitamin D, calcium and the prevention of breast cancer. Breast Cancer Res Treat 121: 469-477. doi:10.1007/s10549-009-0593-9. PubMed: 19851861. 19851861

[69] Gilbert R, Martin RM, Beynon R, Harris R, Savovic J et al. (2011) Associations of circulating and dietary vitamin D with prostate cancer risk: a systematic review and dose-response meta-analysis. Cancer Causes Control 22: 319-340. doi:10.1007/s10552-010-9706-3. PubMed: 21203822. 21203822

[70] Lavie CJ, Lee JH, Milani RV (2011) Vitamin D and cardiovascular disease will it live up to its hype? J Am Coll Cardiol 58: 1547–1556. doi:10.1016/j.jacc.2011.07.008. PubMed: 21958881. 21958881

[71] Anderson JL, May HT, Horne BD, Bair TL, Hall NL et al. (2010) Relation of vitamin D deficiency to cardiovascular risk factors, disease status, and incident events in a general healthcare population. Am J Cardiol 106: 963–968. doi:10.1016/j.amjcard.2010.05.027. PubMed: 20854958. 20854958

[72] Lee JH, O’Keefe JH, Bell D, Hensrud DD, Holick MF (2008) Vitamin D deficiency an important, common, and easily treatable cardiovascular risk factor? J Am Coll Cardiol 52: 1949–1956. doi:10.1016/j.jacc.2008.08.050. PubMed: 19055985. 19055985

[73] Giovannucci E, Liu Y, Hollis BW, Rimm EB (2008) 25-hydroxyvitamin D and risk of myocardial infarction in men: a prospective study. Arch Intern Med 168: 1174–1180. doi:10.1001/archinte.168.11.1174. PubMed: 18541825. 18541825 PMC3719391

[74] Wang TJ, Pencina MJ, Booth SL, Jacques PF, Ingelsson E et al. (2008) Vitamin D deficiency and risk of cardiovascular disease. Circulation 117: 503–511. doi:10.1161/CIRCULATIONAHA.107.706127. PubMed: 18180395. 18180395 PMC2726624

[75] Hsia J, Heiss G, Ren H, Allison M, Dolan NC, et al. (2007) Calcium/vitamin D supplementation and cardiovascular events. Circulation 115: 846-854 (Erratum in: Circulation. 2007 May 15: 115(19): e466) 17309935 10.1161/CIRCULATIONAHA.106.673491

[76] Sugden JA, Davies JI, Witham MD, Morris AD, Struthers AD (2008) Vitamin D improves endothelial function in patients with Type 2 diabetes mellitus and low vitamin D levels. Diabet Med 25: 320–325. doi:10.1111/j.1464-5491.2007.02360.x. PubMed: 18279409. 18279409

[77] Margolis KL, Ray RM, Van Horn L, Manson JE, Allison MA et al. (2008) Effect of calcium and vitamin D supplementation on blood pressure: the Women’s Health Initiative Randomized Trial. Hypertension 52: 847–855. doi:10.1161/HYPERTENSIONAHA.108.114991. PubMed: 18824662. 18824662 PMC2791957

[78] Gepner AD, Ramamurthy R, Krueger DC, Korcarz CE, Binkley N et al. (2012) A prospective randomized controlled trial of the effects of vitamin D supplementation on cardiovascular disease risk. PLOS ONE 7: e36617. doi:10.1371/journal.pone.0036617. PubMed: 22586483. 22586483 PMC3346736

[79] Thacher TD, Clarke BL (2011) Vitamin D insufficiency. Mayo Clin Proc 86: 50–60. doi:10.4065/mcp.2011.0356. PubMed: 21193656. 21193656 PMC3012634

[80] Bouillon R, Eelen G, Verlinden L, Mathieu C, Carmeliet G et al. (2006) Vitamin D and cancer. J Steroid Biochem Mol Biol 102: 156-162. doi:10.1016/j.jsbmb.2006.09.014. PubMed: 17113979. 17113979

[81] Holick MF (2004) Vitamin D: importance in the prevention of cancers, type 1 diabetes, heart disease, and osteoporosis. Am J Clin Nutr 79: 362-371. PubMed: 14985208. 14985208 10.1093/ajcn/79.3.362

[82] van den Bergh JP, Bours SP, van Geel TA, Geusens PP (2011) Optimal use of vitamin D when treating osteoporosis. Curr Osteoporos Rep 9: 36-42. doi:10.1007/s11914-010-0041-0. PubMed: 21113692. 21113692 PMC3026680

[83] Mithal A, Wahl DA, Bonjour JP, Burckhardt P, Dawson-Hughes B et al. (2009) Global vitamin D status and determinants of hypovitaminosis D. Osteoporos Int 20: 1807-1820. doi:10.1007/s00198-009-0954-6. PubMed: 19543765. 19543765

[84] Cashman KD (2012) The role of vitamers and dietary-based metabolites of vitamin D in prevention of vitamin D deficiency. Food. Nutr Res 56:Epub. 10.3402/fnr.v56i0.5383 PMC3321253 22489218

